# Socio-Economic Inequality of Chronic Non-Communicable Diseases in Bangladesh

**DOI:** 10.1371/journal.pone.0167140

**Published:** 2016-11-30

**Authors:** Tuhin Biswas, Md. Saimul Islam, Natalie Linton, Lal B. Rawal

**Affiliations:** 1 Health Systems and Populations Studies Division, icddr,b, Mohakhali, Bangladesh; 2 Oregon State University, Corvallis, Oregon, United States of America; 3 James P Grant Schools of Public Health, BRAC University, Dhaka, Bangladesh; BRAC, BANGLADESH

## Abstract

**Introduction:**

Chronic non-communicable diseases (NCDs) are a major public health challenge, and undermine social and economic development in much of the developing world, including Bangladesh. Epidemiologic evidence on the socioeconomic status (SES)-related pattern of NCDs remains limited in Bangladesh. This study assessed the relationship between three chronic NCDs and SES among the Bangladeshi population, paying particular attention to the differences between urban and rural areas.

**Materials and Method:**

Data from the 2011 Bangladesh Demographic and Health Survey were used for this study. Using a concentration index (CI), we measured relative inequality across pre-diabetes, diabetes, pre-hypertension, hypertension, and BMI (underweight, normal weight, and overweight/obese) in urban and rural areas in Bangladesh. A CI and its associated curve can be used to identify whether socioeconomic inequality exists for a given health variable. In addition, we estimated the health achievement index, integrating mean coverage and the distribution of coverage by rural and urban populations.

**Results:**

Socioeconomic inequalities were observed across diseases and risk factors. Using CI, significant inequalities observed for pre-hypertension (CI = 0.09, p = 0.001), hypertension (CI = 0.10, p = 0.001), pre-diabetes (CI = -0.01, p = 0.005), diabetes (CI = 0.19, p<0.001), and overweight/obesity (CI = 0.45, p<0.001). In contrast to the high prevalence of the chronic health conditions among the urban richest, a significant difference in CI was observed for pre-hypertension (CI = -0.20, p = 0.001), hypertension (CI = -0.20, p = 0.005), pre-diabetes (CI = -0.15, p = 0.005), diabetes (CI = -0.26, p = 0.004) and overweight/obesity (CI = 0.25, p = 0.004) were observed more among the low wealth quintiles of rural population. In the same vein, the poorest rural households had more co-morbidities compared to the richest rural households (p = 0.003), and prevalence of co-morbidities was much higher for the richest urban households compared to the poorest urban households. On the other hand in rural the “disachievement” of health indicators is more noticeable than the urban ones.

**Conclusion:**

The findings indicate the high burden of selected NCDs among the low wealth quintile populations in rural areas and wealthy populations in urban areas. Particular attentions may be necessary to address the problem of NCDs among these groups.

## Introduction

Over the past few decades Bangladesh, a country of over 150 million people, has made has made tremendous progress in achieving health and economic development [1], such as cutting down its maternal mortality ratio [2] and making impressive gains in life expectancy [3]. Nonetheless, the country still faces many public health challenges as it undergoes a demographic and epidemiological transition from dealing primarily with infectious diseases to combating the increasing problem of chronic non-communicable diseases (NCDs) in the context of high socioeconomic inequality [4] and a largely rural population.

In the World Health Organization’s (WHO) Southeast Asia Region, of which Bangladesh is a part, NCDs such as heart disease, stroke, cancer, chronic respiratory diseases and diabetes are estimated to account for half of annual mortality (54%) and nearly half of the burden of disease (47%) [5]. In Bangladesh, NCDs account for 61% of the total disease burden [6], and disease trends suggest that major NCDs such as cardiovascular diseases, diabetes, cancer, and chronic respiratory diseases will impose even larger burdens in the near future. The limited evidence available suggests that NCDs in Bangladesh are responsible for more than half of annual mortality [4]. In 2011, the Bangladesh Health and Demographic Survey (BDHS) found the age-adjusted prevalence of diabetes and pre-diabetes to be 9.7% and 22.4% [7], and the age-adjusted prevalence of pre-hypertension and hypertension were 27.1% and 24.4% [8]. Obesity is also an emerging public health problem in Bangladesh, and an NCD risk factors survey conducted in 2010 found overall prevalence of obesity was 11.6%. The survey also found that 98.7% of Bangladeshis had at least one risk factor for developing NCDs, 77.4% had two or more risk factors, and 28.3% had 3 or more risk factors[9].

Limited resources, a weak public health system, a highly unregulated private health sector, and an aging population also present significant challenges to effectively tackling the growing burden of NCDs in Bangladesh. [10–12] More than 70% of the population currently lives in rural areas, [13] where healthcare resources are most limited, and nearly half of the population subsists on less than US$1.25 per day [14]. Bangladesh spends only 3.5% of its GDP on health, and 63% of all health expenditure is from out-of-pocket expenses [15].

Care and treatment of NCDs long-term plans and most often require high cost than treating communicable diseases, and in country like Bangladesh, the poor have less access to such proper care and even if they are available, the services are quite expensive for NCDs. Tertiary level hospitals, mostly located in major cities, they provide treatment and rehabilitation services for most chronic NCDs such as cardiovascular diseases, diabetes and cancer. However, many public tertiary care hospitals are overloaded and lack adequate infrastructure to meet the service needs of patients—especially those suffering from NCDs. Private hospitals, on the other hand, are expensive, so only the wealthy people are able to utilize services.Gaps in health-related outcomes between the rich and poor are large in developing countries including Bangladesh [16–22]. These gaps have greater potentials for limiting the poor to contributing to the national economy [23]. In this study, we estimated socioeconomic inequality between Bangladeshis with three common chronic NCDs, stratified by urban and rural areas of residence in Bangladesh.

## Methodology

The data for this study were derived from the 2011 Bangladesh Demographic and Health Survey (BDHS). Data collection processes and methodology of the BDHS are described elsewhere [24]. The 2011 BDHS was the sixth iteration of the BDHS, and was the first time a national survey in Bangladesh incorporated measurement of biomarkers for NCDs, such as blood pressure and blood glucose levels. The use of standardized measures (versus self-report) to determine health status promotes better detection of chronic health conditions among those of lower socioeconomic status (SES), as the rural poor are less likely to be objectively screened for NCDs compared to those of higher SES [25–28]. In BDHS 2011`the survey team collected data from 17141 households. One third of the HHs[29] was selected for biomarker test using systematic random sampling. All men and women age 35 years and above were eligible for the biomarker test and total biomarker measures were collected from 8,835 (male: 4524, female: 4311) who were eligible and were available during the time of data collection.

### Measurement of NCDs

Detailed information on the socio demographic characteristics of all participants was collected by trained staff using a standardized questionnaire that also contained questions on the diagnosis and treatment of diabetes and hypertension. Each data collection team included a health technician who was trained to measure blood pressure and collect blood samples. Blood pressure, blood glucose concentration, body weight and height were assessed using standard methods, as previously described[30].Blood pressure was measured using a LifeSource UA-767 Plus blood pressure monitor (A&D Medical, San Jose, USA), as recommended by the World Health Organization (WHO). Three measurements were taken at approximately 10-minute intervals and the respondent’s blood pressure was obtained by averaging the second and third measurements. Blood glucose was measured using the HemoCue Glucose 201 Analyzer (Teleflex Medical L.P., Markham, Canada) in whole blood obtained by finger prick from capillaries in the middle or ring finger after an overnight fastan approach that is widely used in resource-limited countries[31, 32]. Blood glucose measurements were adjusted to obtain equivalent plasma glucose levels[33]. Height and weight were measured at the participant’s home by trained field research staff. Weight was measured twice to the nearest 0.1 kg with light clothing on and without shoes by digital weighing scales placed on a flat surface. The average of the measurements was used in the analysis. Height was measured three times using a standard clinical height scale with patient standing without shoes.

### Measurement of Socioeconomic Status and Inequality

Data reflecting socioeconomic status (SES) were collected in the BDHS using the Demographic and Health Survey wealth index, which relies on ownership of selected assets to determine relative wealth. The wealth index was developed through principle components analysis with data collected in the Household Questionnaire portion of the 2011 BDHS[29]. Household assets were used to construct asset quintiles, and based on these asset quintiles households were placed on a continuous scale of relative wealth from “poorest” to “richest.” Inequality by SES/wealth quintile was then assessed using a concentration index (CI).

### Achievement Index

The mean level of the indicator and the distributional pattern of the indicator, as estimated by the concentration index, can be combined into an index of health achievement. The health achievement index has been calculated for the socioeconomic distribution of all indicators using the measure of “achievement” as proposed by Wagstaff [34]. The larger value of the index is considered as higher health disachievment to one group of population than others group.

### Data Analysis

A CI and its associated curve [35–37] can be used to identify whether SES-related inequality exists for a given health variable [38]. It has been used, for example, to measure and compare the degree of SES-related inequality in child health, adult health, health subsidies, and healthcare utilization [39, 40]. The CI can be defined with a curve, which graphs on the x-axis the cumulative percentage of the sample, ranked by SES, and on the y-axis the corresponding cumulative percentage of the health variables of interest. The index is bounded between -1 and 1. The concentration index is defined as twice the area between the concentration curve and the line of equality, which is set at a 45-degree angle. A CI value of zero means that there is no SES-related inequality. When the values of the CI are negative, the curve lies above the line of equality, indicating that there exists an increased concentration of the health variable among the poor, and a positive values shows the curve lies below the line of equality, indicating the health variable is disproportionately present among the rich. STATA 11 (StataCorp LP) was used for all statistical analyses.

## Results

The general characteristics of participants included in the analysis are presented in **Table 1.**

**Table 1:** General characteristics of adult Bangladeshis by socioeconomic status, 2011 Bangladesh Health and Demographic Survey.

Variables	Poorest	Poorer	Middle	Richer	Richest	Total
**Sex of household member**						
Male	824 (52.5)	820 (52)	850 (50.7)	901 (49.8)	1129 (51.2)	4524 (51.2)
Female	746 (47.5)	757 (48)	826 (49.3)	908 (50.2)	1074 (48.8)	4311 (48.8)
**Age**						
Mean ± SD	51.81±13.2	51.2±13.0	51.7±13.1	51.9±13.5	50.5±12.4	51.3±13.0
**Marital Status**						
Never married	8 (0.7)	1 (0.1)	9 (0.7)	12 (0.9)	16 (1)	46 (0.7)
Married	1183 (99.3)	1159 (99.9)	1226 (99.3)	1329 (99.1)	1579 (99)	6476 (99.3)
**Highest level of education**						
No education	1250 (79.6)	1098 (69.6)	1036 (61.8)	959 (53)	922 (41.9)	5265 (59.6)
Primary	251 (16)	310 (19.7)	404 (24.1)	405 (22.4)	322 (14.6)	1692 (19.2)
Secondary	67 (4.3)	149 (9.4)	193 (11.5)	318 (17.6)	488 (22.2)	1215 (13.8)
College or higher	2 (0.1)	20 (1.3)	43 (2.6)	127 (7)	471 (21.4)	663 (7.5)
**Residence**						
Urban	223 (14.2)	187 (11.9)	329 (19.6)	680 (37.6)	1573 (71.4)	2992 (33.9)
Rural	1347 (85.8)	1390 (88.1)	1347 (80.4)	1129 (62.4)	630 (28.6)	5843 (66.1)

**Fig 1:**
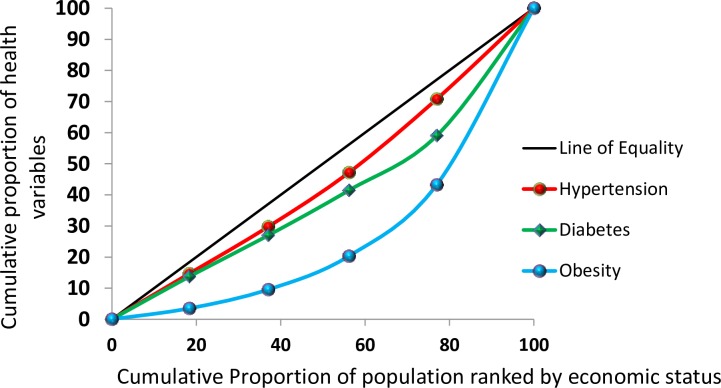
Concentration curve by health condition, 2011 Bangladesh Health and Demographic Survey

**Fig 2:**
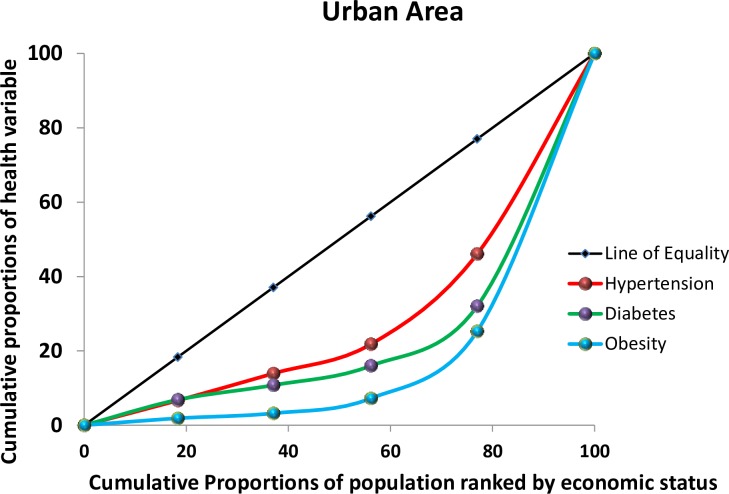
Concentration curve by health condition in urban areas, 2011 Bangladesh Health and Demographic Survey

**Fig 3:**
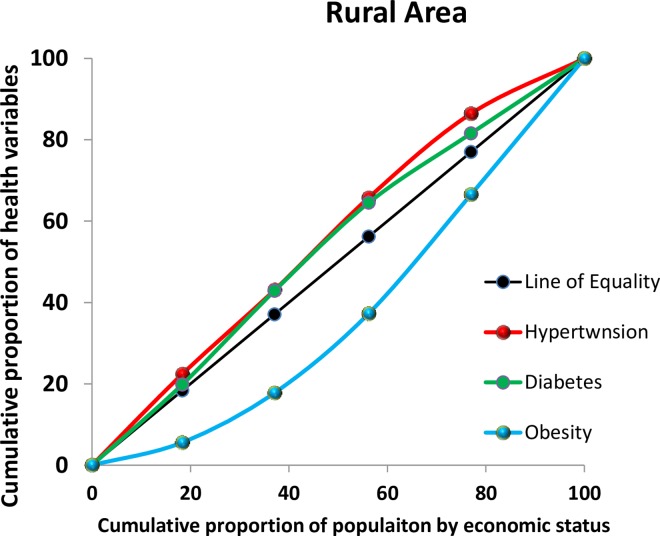
Concentration curve by health condition in rural areas, 2011 Bangladesh Health and Demographic Survey

Overall, hypertension, diabetes, and overweight/obesity were found to be more prevalent among the richest Bangladeshis (**Fig 1)**. However, when stratified by urban and rural area of residence the high prevalence of these conditions among the richest was only observed in urban areas, while in rural areas the health conditions were more prevalent among the poor and poorest (**Fig 2 and Fig 3)**.

Prevalence and CI values for pre-hypertension and hypertension across socioeconomic quintiles are presented in **Table 2**.

**Table 2:** Prevalence of pre-hypertension and hypertension by socioeconomic status (SES) quintile, 2011 Bangladesh Health and Demographic Survey

SES quintile	Normal			Pre-hypertension			Hypertension		
	Urban (%)	Rural (%)	Total (%)	Urban (%)	Rural (%)	Total (%)	Urban (%)	Rural (%)	Total (%)
Poorest	13.5	86.5	20.9	15.5	84.6	14.9	14.3	85.7	14.7
Poorer	12.8	87.2	21.0	9.5	90.5	15.4	13.9	86.1	15.1
Middle	20.0	80.0	20.6	17.4	82.6	18.6	19.0	81.0	17.4
Richer	37.1	62.9	19.7	36.9	63.1	21.5	41.6	58.4	23.6
Richest	65.4	34.6	17.8	71.9	28.1	29.6	71.7	28.3	29.2
Total	28.6	71.4	48.6	36.2	63.8	39.9	38.3	61.7	11.6
Concentration Index (CI)	0.26	-0.21	-0.07	0.36	-0.20	0.09	0.33	-0.20	0.10
SE of CI	0.002	0.002	0.002	0.002	0.005	0.001	0.015	0.017	0.001
P-value	<0.001	<0.001	0.001	0.000	0.001	0.001	0.003	0.005	0.001

SE: Standard error

Overall prevalence of normal blood pressure, pre-hypertension, and hypertension among survey participants were 48.6%, 39.9%, and 11.6% respectively. The prevalences of pre-hypertension (CI = 0.09, p = 0.001) and hypertension (CI = 0.10, p = 0.001) were significantly higher in the richest group compared to the poorest group. However, when stratified by area of residence, pre-hypertension in rural areas was higher in the poorest group compared to the richest group (84.6 v 28.1%), and prevalence of hypertension was three times as high in the poorest rural men and women compared to the rural richest (85.7% v 28.3%). In rural areas, the CI values for pre-hypertension and hypertension in rural areas were large and negative indicating that the conditions were highly concentrated among the poor in rural areas. In contrast, the CI values were large and positive in urban areas, indicating that the conditions were more concentrated among the rich.

Likewise, pre-diabetes (CI = 0.36, p = 0.002) and diabetes (CI = 0.32, p = 0.002) were concentrated among the rich in urban areas (**Table 3**), and in rural areas the prevalence rates of pre-diabetes (CI = -0.15, p = 0.005) and diabetes (CI = -0.26, p = 0.004) were 2–3 times higher in the poorest households compared to the richest households.

**Table 3:** Prevalence of pre-diabetes and diabetes by socioeconomic status (SES) quintile, 2011 Bangladesh Health and Demographic Survey.

SES quintile	Normal			Pre-diabetes			Diabetes		
	Urban (%)	Rural (%)	Total (%)	Urban (%)	Rural (%)	Total (%)	Urban (%)	Rural (%)	Total (%)
Poorest	14.2	85.9	18.08	14.1	85.9	19.3	15.0	85.0	13.7
Poorer	10.9	89.1	18.56	13.7	86.3	18.7	11.3	88.7	13.3
Middle	19.8	80.2	20.16	15.9	84.1	19.9	21.3	78.7	14.5
Richer	39.2	60.8	21.70	32.7	67.3	19.7	38.3	61.7	17.6
Richest	69.7	30.3	21.49	70.1	30.0	22.4	75.1	24.9	41.0
Total	32.1	67.9	59.66	30.6	69.4	24.7	44.2	55.9	15.7
Concentration Index (CI)	0.35	-0.16	-0.007	0.36	-0.15	-0.01	0.32	-0.26	0.19
SE of CI	0.006	0.007	0.001	0.009	0.012	0.001	0.012	0.014	0.001
P-value	0.001	0.003	0.014	0.002	0.005	0.005	0.002	0.004	<0.001

SE: Standard error

**Table 4**depicts the overall prevalence of being underweight (30.3%), normal weight (57.7%), or overweight/obese (12.0%). The prevalence of being underweight was nearly three times as high in the poorest households compared to the richest households (27.2% v 9.7%). Conversely, prevalence of overweight/obesity was three times higher among the richest households compared to the poorest households (12.0% v 3.5%). The CI for underweight and overweight/obesity had opposite signs and high values, indicating that being underweight was highly concentrated among poor (CI = -0.21, p = <0.001) and being overweight was highly concentrated among the rich (CI = 0.45, p = <0.001).

**Table 4:** Prevalence of normal weight, underweight, and overweight/obesity by socioeconomic status (SES) quintile, 2011 Bangladesh Health and Demographic Survey

SES quintile	Normal			Underweight			Overweight/Obesity		
	Urban (%)	Rural (%)	Total (%)	Urban (%)	Rural (%)	Total (%)	Urban (%)	Rural (%)	Total (%)
Poorest	14.0	86.0	16.2	13.9	86.1	27.2	30.4	69.6	3.5
Poorer	9.5	90.5	17.5	13.2	86.8	24.1	12.5	87.5	6.1
Middle	17.0	83.0	19.6	21.3	78.7	22.6	21.1	78.9	10.8
Richer	36.7	63.3	22.7	35.2	64.8	16.4	44.4	55.6	22.9
Richest	69.7	30.3	24.0	53.1	46.9	9.7	74.3	25.7	56.8
Total	32.3	67.7	57.7	22.7	77.3	30.3	56.4	43.6	12.0
Concentration Index (CI)	0.37	-0.17	0.03	0.26	-0.07	-0.21	0.19	-0.25	0.45
SE of CI	0.007	0.009	0.002	0.010	0.012	0.002	0.016	0.017	0.001
P-value	0.001	0.003	0.003	0.002	0.009	<0.001	0.005	0.004	<0.001

SE: Standard error

The poorest rural households also had more comorbidities compared to the richest rural households (CI = -0.59, p = 0.021 for presence of all three health conditions), and prevalence of comorbidities was much higher for the richest urban households compared to the poorest urban households (CI = 0.70, p = 0.009) (**Table 5**).

**Table 5:** Prevalence of hypertension, diabetes, and overweight/obesity by socio-economic status (SES) quintile, 2011 Bangladesh Health and Demographic Survey

SES quintile	One health condition			Two health conditions			Three health conditions		
	Urban (%)	Rural (%)	Total (%)	Urban (%)	Rural (%)	Total (%)	Urban (%)	Rural (%)	Total (%)
Poorest	14.4	85.6	13.0	26.3	73.7	7.1	0.0	0.0	0.0
Poorer	13.4	86.6	13.7	5.3	94.7	7.1	0.0	0.0	0.0
Middle	21.7	78.3	16.1	11.5	88.5	9.67	0.0	0.0	0.0
Richer	40.0	60.0	21.7	45.5	54.5	16.4	50.0	50.0	23.5
Richest	75.0	25.0	35.5	71.4	28.6	59.9	76.9	23.1	76.5
Total	42.5	57.5	88.68	53.5	46.5	10.65	70.6	29.4	0.6
Concentration Index (CI)	0.48	-0.06	0.16	0.62	0.17	0.42	0.70	0.59	0.67
SE of CI	0.005	0.004	0.002	0.018	0.010	0.005	0.108	0.205	0.094
P value	0.001	0.004	0.001	0.002	0.003	0.001	0.009	0.021	0.009

SE: Standard error

**Table 6**describes the findings of health achievement index for pre-hypertension, hypertension, pre-diabetes, diabetes and overweight/obesity by place of residence. The average level of all indicators was higher in the rural areas compared to urban counterparts. In general, raising average value meaning that the level of “disachievement” becomes larger and larger. This “disachievement” of all the indicators is more pronounced in the rural population compared to the urban ones.

**Table 6:** Average level of health condition and health achievement index

Health conditions	Total		Urban		Rural	
	Average/coverage	Health achievement index	Average/coverage	Health achievement index	Average/coverage	Health achievement index
Pre-hypertension	16.2	14.8	5.9	3.7	10.3	12.4
Hypertension	4.6	4.1	1.6	1.1	2.9	3.5
Pre-diabetes	12.2	12.3	3.5	2.2	8.6	9.9
Diabetes	7.4	5.9	3.0	4.0	4.3	5.4
Overweight/Obesity	6.5	3.6	3.1	2.5	2.5	3.1

## Discussion

This paper describes the socioeconomic inequality of three common chronic NCDs stratified by rural or urban area of residence in Bangladesh. Analysis of these groups by CI found that the health conditions were more prevalent among the richest Bangladeshis in urban areas and the poorest Bangladeshis in rural areas. At the same time achievement index also reported that “disachievement” of health related indicators are larger among the rural population compared to the urban counterparts. A report published in 2013 demonstrated that those who live in poor or marginalized communities have a higher risk of dying from non-communicable diseases than more advantaged groups and communities[41]. Another study in ten European counties reported that ischemic heart diseases (IHD) mortality was higher in those with a lower socioeconomic status[42]. In contrast, studies in India reported an increased risk of cardiovascular disease and cardio-metabolic risk factors among the rich [26, 27, 43] and that several NCDs were particularly concentrated among the rich according to self-reported diagnoses[44]. Another study in Southeast Asia also reported that many adverse risk factors of NCDs are concentrated among the poor[45], and a study using 2002–2004 World Health Survey data from 41 low- and middle-income countries demonstrated that wealth and education were inversely associated with different NCDs[46]. Low SES may also increase the risk of mortality due to NCDs [47].

This clear difference in distribution by area of residence indicates the needs to address NCD and NCD risk factor prevention differently in urban and rural areas. Although the study was limited in scope by the cross-sectional nature of the BDHS data, it nevertheless presents a compelling snapshot of the urban-rural divide in NCD risk factors. One clear strength of the BDHS is that it provides standard measurement for the detection of chronic NCDs. Standardized measurements are often unavailable among those with low SES is due a lack of resources to perform the measurements[27].

Treatment of chronic conditions is expensive and can exacerbate household poverty. Poverty increases the risks of developing a chronic disease[48], and our study demonstrated that the rural poor were more likely to have a chronic health condition of interest, as well as indicate a higher burden of disease in rural areas. As most Bangladeshis reside in rural areas, this is an important finding that must be taken into consideration in strategic planning around poverty alleviation and public health promotion [35]. The poor and the disadvantaged in Bangladesh have significantly less access to healthcare services compared to the rich and the privileged [10]—particularly in rural areas where there are fewer healthcare resources. Health insurance is nearly non-existent in Bangladesh, making the accessibility of healthcare services for the poor more problematic.

Further studies on changes of inequality in chronic NCDs over time, as well as on the sociodemographic factors that influence inequality, are needed for us to better understand the underlying causes and reasons for the current distribution of chronic NCDs in Bangladesh. In rural areas, combining national strategies for poverty alleviation with strategies to promote low-cost NCD prevention and management programs can help ameliorate the increasing burden of disease and mortality associated with low SES in rural Bangladesh.

The social and environmental processes that drive this inequitable distribution of disease in Bangladesh have not been explicitly determined, and must continue to be researched in light of the clear presence of differences between urban and rural areas. Previous studies have attempted to identify determinants of socioeconomic inequalities in health status in Bangladesh and its South Asian neighbors, but further research is warranted [49–51].

## Conclusion

We conclude that diabetes, hypertension and obesity are more prevalent among the wealthy in urban areas and the poor in rural areas of Bangladesh. This clear difference indicates the needs for developing targeted intervention approaches to address the growing problem of NCDs and related risk factors among these populations.

## References

[1] Commission BP. Millennium development goals: Bangladesh progress report-2015 Dhaka: Bangladesh Planning Commission 2013.

[2] El Arifeen S, Hill K, Ahsan KZ, Jamil K, Nahar Q, Streatfield PK. Maternal mortality in Bangladesh: a Countdown to 2015 country case study. The Lancet. 2014;384:1366–74. 10.1016/S0140-6736(14)60955-7 24990814

[3] Chowdhury ME, Ahmed A, Kalim N, Koblinsky M. Causes of maternal mortality decline in Matlab, Bangladesh. Journal of Health, Population and Nutrition. 2009:108–23. 10.3329/jhpn.v27i2.3325 PMC2761779 19489410

[4] Bleich SN, Koehlmoos TL, Rashid M, Peters DH, Anderson G. Noncommunicable chronic disease in Bangladesh: overview of existing programs and priorities going forward. Health Policy. 2011;100:282–9. 10.1016/j.healthpol.2010.09.004 20889225 PMC3043199

[5] Organization WH. Global health risks: mortality and burden of disease attributable to selected major risks: World Health Organization; 2009.

[6] Organization WH. Non-Communicable Disease Risk Factor Survey, Bangladesh 2010 Dhaka: WHO; 2011.

[7] Akter S, Rahman MM, Abe SK, Sultana P. Prevalence of diabetes and prediabetes and their risk factors among Bangladeshi adults: a nationwide survey. Bull World Health Organ. 2014;92:204–13A. 10.2471/BLT.13.128371 24700980 PMC3949596

[8] Rahman MM, Gilmour S, Akter S, Abe SK, Saito E, Shibuya K. Prevalence and control of hypertension in Bangladesh: a multilevel analysis of a nationwide population-based survey. Journal of hypertension. 2014. 10.1097/HJH.0000000000000421 25380166

[9] Organization WH. Non-Communicable Disease Risk Factor Survey Bangladesh 2010. Bangladesh: WHO Press; 2011.

[10] Islam A, Biswas T. Health System Bottlenecks in Achieving Maternal and Child Health-Related Millennium Development Goals: Major Findings from District Level in Bangladesh.

[11] Islam A, Biswas T. Health System in Bangladesh: Challenges and Opportunities. American Journal of Health Research. 2014;2:366–74.

[12] Islam A, Biswas T. Bangladesh Health System and the Millennium Development Goals: Strategic Policy Options for Sustained Progress in Maternal and Child Health. European Journal of Preventive Medicine. 2015;3:63–70.

[13] Baker JL. Dhaka: Improving living conditions for the urban poor. 2007.

[14] (BBS). BBoS. tatistical pocketbook of Bangladesh 2010. Dhaka: BBS. 2011.

[15] Islam A, Ahsan G, Biswas T. Health System Financing in Bangladesh: A Situation Analysis.

[16] Gwatkin DR. Health inequalities and the health of the poor: What do we know? What can we do? Bulletin of the world health organization. 2000;78:3–18. 10686729 PMC2560590

[17] Baker J, van der Gaag J. Equity in health care and health care financing: evidence from five developing countries. Equity in the finance and delivery of health care: An international perspective. 1993.

[18] Leon DA, Walt G. Poverty, inequality, and health: an international perspective: Oxford University Press; 2001.

[19] Wagstaff A. Inequalities in health in developing countries: swimming against the tide?: World Bank Publications; 2002.

[20] Wagstaff A. Poverty and health sector inequalities. Bulletin of the world health organization. 2002;80:97–105. 11953787 PMC2567730

[21] Wagstaff A. Research on equity, poverty and health outcomes: lessons for the developing World. 2000.

[22] Ruger JP, Kim H-J. Global health inequalities: an international comparison. Journal of epidemiology and Community Health. 2006;60:928–36. 10.1136/jech.2005.041954 PMC2465481 17053281

[23] Ahmed S, Hasan MM, Ahmed W, Chowdhury MAH. Socio-economic Inequity of Malnutrition among under-five Children and Women at Reproductive Age in Bangladesh. Journal of Nutrition and health. 2013;1:13–7.

[24] DHS M. Demographic and Health survey. 2012.

[25] Gupta R, Guptha S, Joshi R, Xavier D. Translating evidence into policy for cardiovascular disease control in India. Health Res Policy Syst. 2011;9. 10.1186/1478-4505-9-8 PMC3045991 21306620

[26] Gupta R, Gupta V, Sarna M, Prakash H, Rastogi S, Gupta K. Serial epidemiological surveys in an urban Indian population demonstrate increasing coronary risk factors among the lower socioeconomic strata. JOURNAL-ASSOCIATION OF PHYSICIANS OF INDIA. 2003;51:470–8. 12974428

[27] Reddy KS, Prabhakaran D, Jeemon P, Thankappan K, Joshi P, Chaturvedi V, et al Educational status and cardiovascular risk profile in Indians. Proceedings of the National Academy of Sciences. 2007;104:16263–8. 10.1073/pnas.0700933104 PMC2042195 17923677

[28] Zaman MJ, Patel A, Jan S, Hillis GS, Raju PK, Neal B, et al Socio-economic distribution of cardiovascular risk factors and knowledge in rural India. International journal of epidemiology. 2012:dyr226. 10.1093/ije/dyr226 22345313

[29] Demographic B. Health survey BDHS (2011). NIPORT, Dhaka, Bangladesh. 2013.

[30] Demographic B. Health Survey 2011. Ministry of Health and Population, Government of Nepal. Kathmandu.[online] Available at: http://www.measuredhs.com/pubs/pdf/FR257/FR257 [13April2012]. pdf. 2012.

[31] Sayeed MA, Banu A, Khan AR, Hussain MZ. Prevalence of diabetes and hypertension in a rural population of Bangladesh. Diabetes care. 1995;18:555–8. 7497870 10.2337/diacare.18.4.555

[32] Organization WH. Definition and diagnosis of diabetes mellitus and intermediate hyperglycaemia: report of a WH. 2006.

[33] D’Orazio P, Burnett RW, Fogh-Andersen N, Jacobs E, Kuwa K, Külpmann WR, et al Approved IFCC recommendation on reporting results for blood glucose (abbreviated). Clinical chemistry. 2005;51:1573–6. 10.1373/clinchem.2005.051979 16120945

[34] Wagstaff A. Inequality aversion, health inequalities and health achievement. Journal of health economics. 2002;21:627–41. 12146594 10.1016/s0167-6296(02)00006-1

[35] Kakwani NC. Measurement of tax progressivity: an international comparison. The Economic Journal. 1977:71–80.

[36] Kakwani NC, Bank W. Income inequality and poverty: methods of estimation and policy applications: Oxford University Press New York; 1980.

[37] Kakwani N, Wagstaff A, Van Doorslaer E. Socioeconomic inequalities in health: measurement, computation, and statistical inference. Journal of econometrics. 1997;77:87–103.

[38] O'Donnell OA, Wagstaff A. Analyzing health equity using household survey data: a guide to techniques and their implementation: World Bank Publications; 2008.

[39] Wagstaff A. The bounds of the concentration index when the variable of interest is binary, with an application to immunization inequality. Health economics. 2005;14:429–32. 10.1002/hec.953 15495147

[40] Wagstaff A, Van Doorslaer E. Measuring inequalities in health in the presence of multiple‐category morbidity indicators. Health economics. 1994;3:281–91. 7994327 10.1002/hec.4730030409

[41] Di Cesare M, Khang Y-H, Asaria P, Blakely T, Cowan MJ, Farzadfar F, et al Inequalities in non-communicable diseases and effective responses. The Lancet. 2013;381:585–97. 10.1016/S0140-6736(12)61851-0 23410608

[42] Avendano M, Kunst AE, Huisman M, Lenthe FV, Bopp M, Regidor E, et al Socioeconomic status and ischaemic heart disease mortality in 10 western European populations during the 1990s. Heart. 2006;92:461–7. 10.1136/hrt.2005.065532 16216862 PMC1860902

[43] Gupta R, Kaul V, Agrawal A, Guptha S, Gupta V. Cardiovascular risk according to educational status in India. Preventive medicine. 2010;51:408–11. 10.1016/j.ypmed.2010.08.014 20817021

[44] Vellakkal S, Subramanian S, Millett C, Basu S, Stuckler D, Ebrahim S. Socioeconomic inequalities in non-communicable diseases prevalence in India: disparities between self-reported diagnoses and standardized measures. PloS one. 2013;8:e68219 10.1371/journal.pone.0068219 23869213 PMC3712012

[45] Siegel KR, Patel SA, Ali MK. Non-communicable diseases in South Asia: contemporary perspectives. British medical bulletin. 2014;111:31–44. 10.1093/bmb/ldu018 25190759 PMC4416117

[46] Hosseinpoor AR, Bergen N, Mendis S, Harper S, Verdes E, Kunst A, et al Socioeconomic inequality in the prevalence of noncommunicable diseases in low-and middle-income countries: results from the World Health Survey. BMC public health. 2012;12:1. 22726343 10.1186/1471-2458-12-474 PMC3490890

[47] Sommer I, Griebler U, Mahlknecht P, Thaler K, Bouskill K, Gartlehner G, et al Socioeconomic inequalities in non-communicable diseases and their risk factors: an overview of systematic reviews. BMC public health. 2015;15:1. 26385563 10.1186/s12889-015-2227-y PMC4575459

[48] Le Gales-Camus C, Beaglehole R, Epping-Jordan J. Preventing chronic diseases: a vital investment. Geneva: World Health Organization 2005.

[49] Giashuddin M, Kabir M, Hasan M. Economic disparity and child nutrition in Bangladesh. The Indian Journal of Pediatrics. 2005;72:481–7. 15985736 10.1007/BF02724424

[50] Gwatkin D, Rutstein S, Johnson K, Suliman E, Wagstaff A, Amouzou A. Socio-economic differences in health, nutrition, and population within developing countries: An overview. World Bank. Government of the Netherlands, Swedish International Development Agency2007. 2009. 18293634

[51] Chalasani S. Understanding wealth-based inequalities in child health in India: a decomposition approach. Social Science & Medicine. 2012;75:2160–9. 22980028 10.1016/j.socscimed.2012.08.012

